# An unexpected presentation of very severe hypertriglyceridemia in a boy with Coffin-Lowry syndrome: a case report

**DOI:** 10.1186/s12887-023-04376-5

**Published:** 2023-10-28

**Authors:** Sue Lyn Tan, Muhammad Ghazali bin Ahmad Narihan, Ai Jiun Koa

**Affiliations:** 1 Department of Paediatrics, Faculty of Medicine and Health Sciences, Universiti Malaysia Sarawak, Jalan Datuk Muhammad Musa, Kota Samarahan, Sarawak 94300 Malaysia; 2 Department of Paediatrics, Sarawak General Hospital, Jalan Hospital, Kuching, Sarawak, 93586 Malaysia; 3 Department of Radiology, Faculty of Medicine and Health Sciences, Universiti Malaysia Sarawak, Jalan Datuk Muhammad Musa, Kota Samarahan, Sarawak 94300 Malaysia

**Keywords:** Hypertriglyceridemia, Coffin-Lowry syndrome, Intrauterine growth restriction, Lipaemic plasma

## Abstract

**Background:**

Coffin-Lowry syndrome (CLS) is a rare X-linked condition with intellectual disability, growth retardation, characteristic facies and skeletal anomalies. To date, hypertriglyceridemia has not been reported in literature to be associated with CLS.

**Case Presentation:**

Herein, we report a case of very severe hypertriglyceridemia 32 mmol/L (2834 mg/dL) detected incidentally at three months old in an otherwise well boy born late preterm with intrauterine growth restriction, when he presented with lipaemic plasma. He was later diagnosed with CLS. No pathogenic mutations were found for hypertriglyceridemia, and no secondary causes could explain his very severe hypertriglyceridemia.

**Conclusions:**

The very severe hypertriglyceridemia in this case may appear to be a serious presentation of an unrecognised clinical feature of CLS, further expanding its phenotype.

## Background

CLS is a rare but increasingly recognised X-linked semi-dominant syndrome characterised by psychomotor and growth retardation, facial dysmorphism, digit abnormalities and progressive skeletal changes resulting from mutations in the RPS6KA3 gene [1]. They may also have neurological, ophthalmological, hearing, dental, cardiovascular and respiratory problems [1, 2]. Despite its multisystemic manifestations, hypertriglyceridemia (HTG) has not been reported in CLS. HTG is defined as triglyceride (TG) concentrations > 150 mg/dL [3]. It is incredibly uncommon in infancy and, if present, should prompt consideration of familial lipoprotein lipase deficiency [3]. Here, we describe a case of very severe HTG in infancy with no identifiable primary nor secondary cause. The child was subsequently diagnosed with CLS.

## Case presentation

Our patient’s mother was 37 years old at delivery with uncomplicated antenatal history. Our patient was born vigorous via elective lower segment caesarean section at 35 weeks for intrauterine growth restriction (IUGR) with a low birth weight of 2.1 kg. He was admitted for observation in view of prematurity and did not receive any medications. He was given phototherapy for neonatal jaundice and was discharged well on day five of life.

Subsequently, he developed prolonged jaundice and was on serial monitoring of serum bilirubin levels, which were down-trending to 156 µmol/L at two months of age. No sampling issues were reported. However, follow-up blood-taking at three months of age unexpectedly showed a milky appearance of the blood. A repeated sample after four hours of fasting revealed very severe HTG 32 mmol/L (< 1.68 mmol/L) or 2834 mg/dL (< 150 mg/dL). Apart from improving jaundice, he was otherwise well, exclusively breastfed, not on any medications and did not have eruptive xanthomas nor signs of pancreatitis. His liver was just palpable 1 cm below the subcostal margin. Investigations were notable for raised total cholesterol 8.1 mmol/L, aspartate aminotransferase 87 U/L and gamma-glutamyl transpeptidase 374 U/L (Table 1). Hepatic steatosis was seen on ultrasound. There is no parental consanguinity and both parents’ TG levels are normal.

**Table 1:** Laboratory results at different ages and the child’s diet

Age	3 m	4 m	6 m	8 m	1 y 8 m	2 y 8 m	3 y 4 m
**Diet**	Breast-feeding	Portagen	50% Portagen, 50% normal diet	Normal diet	50% Portagen, 50% normal diet	Normal diet	Normal diet
**Lipid Profile**							
Total cholesterol (< 5.2 mmol/L)	8.1	3.85	3.75	3.14	4.6	4.5	4.55
Triglycerides (< 1.68 mmol/L)	32	2.55	2.4	2.66	1.8	2.52	1.22
HDL (> 1.03 mmol/L)	0.45	0.96	0.92	0.81	0.87	0.97	0.93
LDL (< 2.58 mmol/L)	n/a	1.73	1.74	1.12	2.9	2.4	3.07
**Liver Function Test**							
Total bilirubin (3–18 µmol/L)	155	7	6	6	2.9	6.2	< 2.5
Direct bilirubin (< 5 µmol/L)	n/a	2.6	2.5	1.6	< 1.8	2.4	1.7
AST (15–55 U/L)	87	33	38	56	28	29	n/a
ALT (5–45 U/L)	24	19	23	31	14	12	10
Total protein (44–76 g/L)	n/a	62	63	70	73	72	78
Albumin (38–54 g/L)	n/a	47	43	49	48	46	47
Globulin (17–35 g/L)	n/a	15	20	21	25	26	31
ALP (1-449 U/L)	n/a	202	231	305	172	241	168
GGT (8–90 U/L)	374	119	126	152	n/a	n/a	n/a
**Renal Profile**							
Sodium (129–143 mmol/L)	135			138			137
Potassium (3.6–5.8 mmol/L)	5.0			4.2			4.1
Chloride (93–112 mmol/L)	105			103			100
Urea (1.0-6.4 mmol/L)	1.8			3.3			2.3
Creatinine (60–120 µmol/L)	16			30			39

m months, y years, n/a not available

Breast milk was substituted with Portagen formula, a milk protein-based powder with medium chain TG. Dietary medium-chain TG is directly absorbed into the portal vein. They do not require transport in chylomicrons, so TG levels will not increase. TG levels improved remarkably with this dietary modification. At approximately two years old, he could return to a regular diet with decent TG levels (Table 1). In view of IUGR, he was given general advice to adopt a healthy lifestyle, including a low-fat diet, body weight optimisation, smoking avoidance and regular exercise.

**Fig. 1:**
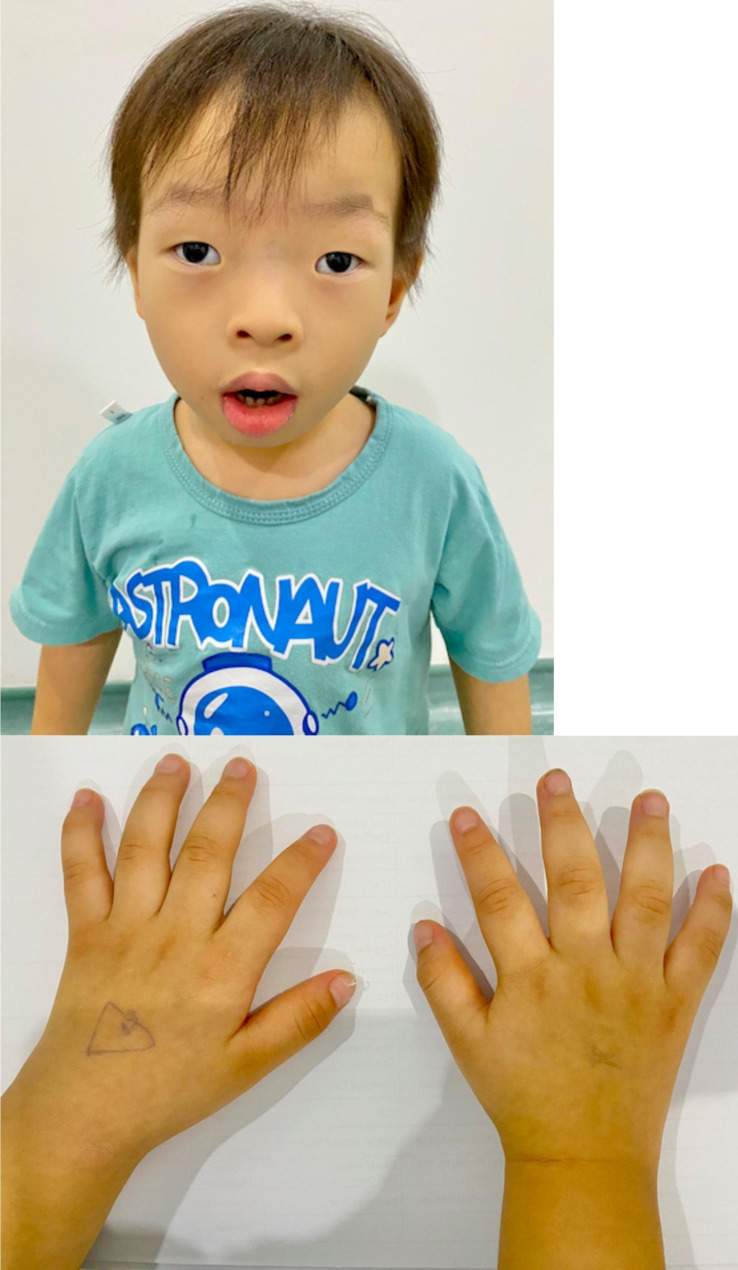
Clinical features of our patient with Coffin-Lowry syndrome: hypertelorism, flat nasal bridge, full lips with eversion of lower lip and fleshy tapering fingers

Over the next 1–2 years of life, our patient was found to have global developmental delay and dysmorphic features (Fig. 1) consistent with CLS. He has hypertelorism, downslanting palpebral fissures, frontal bossing, prominent brows, flat nasal bridge, maxillary hypoplasia, prominent nasal tip, thick nasal septum, thick lower lip, pectus carinatum, tapering fingers and fleshy palms. His growth and hearing assessment was normal. Brain MRI demonstrated generalized reduced white matter volume at bilateral cerebral hemispheres with mild ventriculomegaly.

Eventually, whole exome sequencing (Table 2) was sent and revealed a hemizygous mutation in the RPS6KA3 gene c.748G > A (p.Asp250Asn), confirming CLS. KCNQ1 variant was also reported. Our patient has been reviewed by a cardiologist and so far does not demonstrate any features of associated disease. DNAH9, SP110 and SPATA7 were additional variants found for autosomal recessive conditions, not related to our patient’s phenotype. Despite having multiple pathogenic genes detected, no pathogenic mutations for HTG were found.

**Table 2:** Whole exome sequencing results

Gene	RPS6KA3	KCNQ1
**Classification**	Uncertain significance	Likely pathogenic
**Exon/ Intron**	9	13
**DNA change**	c.748G > A	c.1615 C > T
**Protein change**	p.Asp250Asn	p.Arg539Trp
**Zygosity**	Hemizygous	Heterozygous
**Inheritance**	X-linked dominant	Autosomal dominant; autosomal recessive
**OMIM**	300075	607542
**Associated disease**	Coffin-Lowry syndrome, Intellectual disability, X-linked 19	Atrial fibrillation, familial, Jervell and Lange-Nielsen syndrome, Long QT syndrome, Short QT syndrome

Genetic counselling has been given but parents are not keen for further testing. They are asymptomatic and working as professionals full-time. Clinically, they are of average adult height and do not exhibit any dysmorphic features nor gross skeletal abnormalities characteristic of CLS.

## Discussion and conclusions

CLS is a rare condition with an estimated incidence of 1:50 000 to 1:100 000 [1]. Its X-linked semi-dominant inheritance typically causes males to be severely affected, with females being much more mildly and variably affected [1]. Approximately 70–80% of patients are sporadic cases [1]. It is a well-established syndrome characterised by psychomotor and growth retardation, facial dysmorphism, digit abnormalities and progressive skeletal changes resulting from mutations in the RPS6KA3 gene [1]. Beyond infancy, the diagnosis of the affected male is suspected based on distinctive facial features [2] such as hypertelorism, flat nasal bridge, and full lips with eversion of the lower lip [1, 2, 4], which are seen in our patient. He also has tapering and fleshy digits, one of CLS’s most characteristic features [1, 2, 4]. Radiological signs described include cranial hyperostosis, kyphoscoliosis, and tufted distal phalanges [4, 5]. Generalized reduced brain volume, as seen in our patient, has been reported in a morphometric study of brains in CLS patients [6]. Some uncommonly associated manifestations include sensorineural hearing loss [1], cataracts [5], stimulus-induced drop attacks [7] and mitral valve dysfunction [8]. As aforementioned, most of the literature on CLS focused on physical and radiological features. Despite its protean manifestations, which are markedly variable in severity, biochemical abnormalities have not been reported widely and remain to be discovered. A recent article described a possible biochemical abnormality which is transient hypercalcemia in a girl later found to have CLS [9]. Very severe HTG as seen in our patient, has not been reported in CLS so far.

Overall, our patient’s clinical features are consistent with CLS except for the history of HTG. HTG is defined as TG concentrations > 150 mg/dl [3]. Levels > 2000 mg/dl as seen in our patient is considered very severe [3]. The risk of pancreatitis with levels > 2000 mg/dl is 10–20% [3]. Persistent HTG may also increase the risk of premature cardiovascular disease [3]. Generally, the causes of HTG can be categorised into primary or genetic and secondary causes. It is an extremely uncommon disorder in infancy. TG levels beyond 500 mg/dl are also rare (< 0.2%) [3], and especially when found in an infant should prompt consideration of a primary TG metabolism, particularly familial lipoprotein lipase deficiency [3]. Similarly, lipoprotein lipase deficiency was suspected given the milky appearance of his blood during routine examination. This milky appearance, also known as lipaemic plasma, results from TG accumulation secondary to impaired clearance of chylomicrons from plasma. However, no pathogenic mutations for HTG were found in our patient’s whole exome sequencing. Transient infantile hypertriglyceridemia was also considered, but there were no reportable variants in the glycerol-3-phosphate dehydrogenase (GPD1) gene for this patient. Secondary causes such as diabetes, obesity, metabolic syndrome, hypothyroidism and renal disease [3, 10] were ruled out in our patient, and he was not on any medications such as steroids, estrogen, beta-blockers and antipsychotics [3, 10] that could elevate TG levels. Furthermore, CLS is not known to cause renal dysfunction which could contribute to HTG, and his renal function remained normal. He also did not receive any intravenous lipid, such as those given with parenteral nutrition in small preterm babies. Our patient’s mechanism for HTG is puzzling as it neither fits any of these primary nor secondary causes.

Our patient was preterm and born intrauterine growth restricted. This in itself may be a plausible contribution to his HTG. Preterm infants are at higher risk of HTG than term infants due to their relatively limited muscle and fat mass and, therefore, the decreased hydrolytic capacity of the enzyme lipoprotein lipase [11]. Many studies have documented that IUGR newborns have significantly higher TG levels compared to those appropriate for gestational age newborns [12, 13]. This could be explained by impaired clearance of TG-rich lipoproteins as well as resistance to the action of insulin on lipoprotein lipase in peripheral tissues in IUGR newborns [12]. However, the TG levels in the IUGR infants in these studies (90.23 +/- 48.16 mg/dl [12], 101.14 +/- 24.69 mg/dl [13]), despite being significantly higher than their appropriate for gestational age counterparts, were still well within normal range. Levels as high as our patient is unheard of in IUGR infants.

Given our patient’s diagnosis of CLS and considerable clinical, laboratory, and genetic evidence against another cause and/or second genetic condition, we hypothesise that his transient, yet very severe, HTG in infancy is related to his diagnosis of CLS.

Of note, the patient’s gene for CLS was classified as variant of uncertain significance. This may be attributed in part to the rarity of this syndrome, with only over 200 cases reported worldwide [14]. Also, he is Malaysian, where genomic information on the Asian population is lacking. Furthermore, our primary indication for genetic testing was the presence of hypertriglyceridemia, which is not known to be a feature of CLS. We believe that his variant is de novo since majority of cases are sporadic [1] and his mother has normal intellect and does not manifest any features. Among others, family segregation studies may provide some evidence for reclassification, but without parental consent, this is our limitation.

In conclusion, our patient was found to have transient HTG when he presented with a lipaemic blood sample at three months old. He was diagnosed with CLS about 1 year later. In the absence of any primary and secondary causes of HTG, it is possible that the patient represents a severe presentation of an unrecognised phenotype of CLS that resolves with age and is likely prior to most patients’ diagnosis. Early detection is vital, as very severe HTG may result in devastating consequences like atherosclerosis, pancreatitis and pancreatic necrosis. As genetic testing becomes increasingly offered to patients, more individuals with CLS will be diagnosed in infancy. Earlier diagnosis and phenotyping of individuals with CLS will not only aid in medical management. In addition, they will also help to clarify if HTG, as seen in this case, represents an expansion of the phenotype or a truly unrelated feature.

## Data Availability

Not applicable.

## Abbreviations

CLS: Coffin-Lowry syndrome
HTG: hypertriglyceridemia
TG: triglyceride
IUGR: intrauterine growth restriction
